# Supplementary material for: DepoScope: Accurate phage depolymerase annotation and domain delineation using large language models
Source: PLoS Comput Biol. 2024 Aug 5;20(8):e1011831. doi: 10.1371/journal.pcbi.1011831 (PMC11326577; doi:10.1371/journal.pcbi.1011831)
Supplement: S1 Fig — ESM-2 embedding representations were computed for each PD domain fold of the training dataset. (DOCX) [file pcbi.1011831.s001.docx]

### **Supplementary figure**


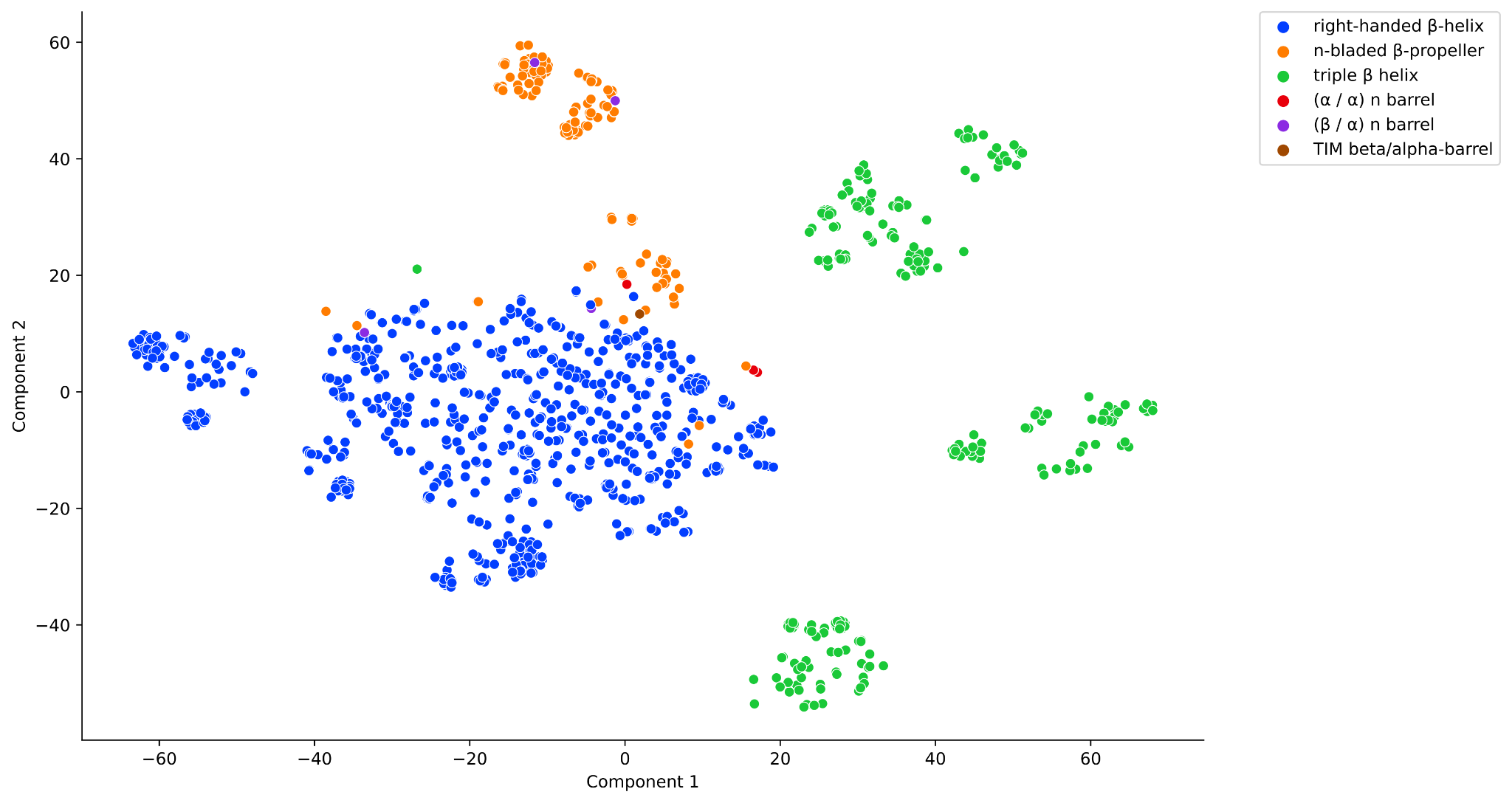


**Supplementary Figure 1**: t-SNE representation of the PD domain fold. ESM-2 embedding representations were computed for each PD domain fold of the training dataset.
