# Supplementary material for: DepoScope: Accurate phage depolymerase annotation and domain delineation using large language models
Source: PLoS Comput Biol. 2024 Aug 5;20(8):e1011831. doi: 10.1371/journal.pcbi.1011831 (PMC11326577; doi:10.1371/journal.pcbi.1011831)
Supplement: Attachment — Submitted filename: RCE_Deposcope_response.pdf [file pcbi.1011831.s005.pdf]

Response to reviewers

DepoScope: accurate phage depolymerase annotation and domain delineation using large language models

We sincerely thank the reviewers for providing us with valuable feedback to further improve our work. Their comments and our point-by-point responses to address their comments are elaborated below.

Reviewer 1

In this study, the authors introduce DepoScope, an innovative machine-learning tool to provide an accurate annotation and domain identification of phage depolymerases. They developed DepoScope using the cutting-edge, pre-trained ESM2 protein language model and a two-layer convolutional neural network. To better train the model, the authors collected and processed a comprehensive set of protein sequences. The findings enhance our understanding of phage depolymerases, especially at the amino acid level. However, some major and minor issues still need to be addressed.

Major comments

1. **The authors have claimed that their model surpasses two other recently developed depolymerase detection tools, DePP and PhageDPO, in performance as evidenced by the benchmark using the dataset from Pires *et al.* My primary concern centers on whether this superior performance of DepoScope is attributed to the curated dataset used for its training or to the inherent strengths of its model architecture. This leads to a crucial question: if DePP and PhageDPO were trained using the same dataset as DepoScope, would their performance metrics, specifically in terms of F1 score and MCC, align closely with those of DepoScope?**

How good or bad a model performs is always a result of both the model architecture and the dataset it has been used to train the model. As we argue in our manuscript, we believe that the dataset we constructed is superior to those constructed by the authors of DePP and PhageDPO (see our answer to comment 2). In this regard, training their models on our dataset could bring the performance of their models closer to ours, but this would simply involve comparing a Random Forest (DePP), Support Vector Machine (PhageDPO) or fully connected neural network (PhageDPO) architecture to our ESM-2 based model architecture. In our view, such a comparison is not relevant because we designed DepoScope from the outset to do both binary classification and token-level classification (which the other model architectures are not able to do). In addition, the training code for PhageDPO is not available (nor does the manuscript contain the appropriate details), practically impeding us from training PhageDPO on our dataset. Instead, we compare the entire approaches followed by the researchers that developed DePP and PhageDPO against our approach, including the data collection and processing. In our view, this is the most relevant comparison if we consider researchers who could be interested in actually using the tool.

2. **The authors have noted that previous studies may have been constrained by "small and undiversified" datasets. However, upon examining the PhageDPO more closely, its training data comprises 1,437 positive sequences from NCBI, encompassing six depolymerase-associated domains. Could the authors kindly provide a comparison between the dataset used in their study (1,926 positive sequences) and that used by PhageDPO?**

We appreciate the reviewer's interest in comparing our dataset with that of PhageDPO. To contrast the datasets used in our research and PhageDPO's, we would like to highlight three main factors that suggest potential constraints in the size and variety of PhageDPO's dataset, which our dataset may not share:

1. Interproscan Entries: the six PhageDPO depolymerase-associated domains now correspond to five unique Interproscan entries (IPR011050, IPR012334, IPR024430, IPR024428, IPR013830), which are only a small subset of the 221 entries we used to create the HMM profile database.

2. Data Filtration Process: our dataset underwent stringent filtration stages, including CD-HIT clustering at 95% identity and affinity propagation clustering. Initially, our dataset comprised 11,513 proteins, which resulted in 6,816 protein clusters after CD-HIT clustering. We followed the data collection processes described in the supplementary data of the PhageDPO paper and managed to collect an initial set of 1,660 sequences. After clustering with CD-HIT at 95%, the PhageDPO dataset yielded 913 protein clusters. This substantial difference indicates potential constraints in the diversity and scale of PhageDPO's dataset compared to ours.

3. Biased Representation: evaluations conducted by the DePP authors suggest a preference by PhageDPO for phages isolated in certain bacterial taxa, implying an underrepresentation of depolymerases from phages isolated from other bacterial taxa. For example, while phages isolated in Cellulophaga and Streptococcus ranked well in PhageDPO, those isolated in Enterobacter, Prochlorococcus, or Streptomyces were not as effectively processed. This discrepancy further underscores potential limitations in the diversity and representativeness of PhageDPO's dataset across various bacterial taxa.

We have extended our manuscript text in the following ways:

Lines 69-71:
"However, a limitation of both tools is the availability of only small or undiversified datasets to train their models (using only a limited number of depolymerase-associated protein domains or only experimentally validated sequences)."

Lines 268-269:
"A total of 11,513 proteins presented a significant hit against a custom PD domain database (comprising sequences across 221 InterPro entries)."

3. **Data imbalance presents a significant challenge in classification tasks. Could the authors consider experimenting with varying numbers of negative controls in their training dataset, similar to the approach taken by PhageDPO, to see how these alterations impact the overall model performance?**

We consider that data imbalance may present a challenge in classification, but not in all cases. Here, we do not train on a completely balanced dataset for two reasons. First, we do not view the current imbalance as problematic (984 positive instances versus 1,409 negative instances); *i.e.*, it is not an extreme imbalance that would prevent the model from learning predictive patterns in any of the classes. For our negative dataset, we specifically collected other proteins involved in catalytic activity and structural proteins without catalytic activity, as these would be potentially hard to correctly classify as negatives. We found 1,409 such proteins. We don't believe that adding other random proteins as negatives or discarding collected ones would be of extra value for the model. Second, despite this imbalance, the model reaches a satisfactory performance across various performance metrics. If this were not the case, we would be more inclined to consider balancing our dataset to potentially improve performance, but we do not believe that training on an imbalanced dataset has fundamental shortcomings. We have adapted our explanation in the text:

Lines 224-227:
"We specifically chose to collect other proteins that are involved in catalytic activity and structural proteins without catalytic activity, as those proteins would be potentially hard for the model to correctly classify as negatives (Supplementary Table 4). In total, 1,409 proteins were collected that matched the annotations of interest and these were added to the training dataset as negative instances. As the number of sequences in the positive and negative classes do not differ substantially, we have not artificially balanced the training dataset."

4. **The authors' decision to exclude certain fold types due to their limited occurrence is understandable. However, in the interest of enhancing the model's generalizability, would it be feasible for the authors to classify folds like the TIM β/α-barrel, and α/α toroid folds under an "others" category?**

We appreciate the reviewer's suggestion aimed at enhancing the model's relevance. However, due to the limited number of occurrences of the TIM β/α-barrel and α/α toroid (totalling no more than 25 instances), creating a robust category would necessitate additional manual effort. Considering the low prevalence of these folds, the benefit of such classification is not evident.

5. **The substantial difference in MCC between DepoScope and the other two models was quite striking. This leads me to question whether the training data for DepoScope included the dataset from Pires et al., especially considering that INPHARED downloads and filters phage genomes from GenBank. For clarity and thorough understanding, it would be beneficial if the authors could explicitly state whether the Pires et al. dataset was incorporated into the training data.**

This is a very good remark by the reviewer. Interestingly, the Pires *et al*. dataset predates any of the depolymerase prediction tools. The dataset was published in 2016, while PhageDPO and DePP were both published in 2023. In fact, upon further analysis, we observe that the datasets used to train PhageDPO and DePP respectively comprise 32% (49 out of 155) and 5% (9 out of 155) of the depolymerases in the Pires *et al*. dataset. Our training dataset comprised 18% (28 out of 155) of the Pires *et al*. dataset. When combined, 78 out of 155 sequences were present in at least one of the training datasets of the benchmarked tools. To mitigate this bias in the benchmark evaluation, we opted to remove these sequences and recompute the benchmark results. Our updated results are in Table 2 and the text (*Lines 331-345*). We have also extended our manuscript text accordingly:

Lines 254-255:
"To mitigate biases, sequences present in the training dataset of either of the tools were not considered."

Updated Table 2:

Models	precision	recall	specificity	accuracy	F1	MCC	PR AUC
DePP	3.5%	**91.6%**	60.9%	61.4%	6.7%	0.131	10.2%
PhageDPO	6.0%	74.7%	82.0%	81.9%	11.2%	0.178	27.8%
DepoScope	**32.0%**	69.0%	**98.0%**	**97.2%**	**43.4%**	**0.455**	**42.3%**
DepoScope FP adjusted	81.4%	69.0%	99.8%	99.3%	74.5%	0.744	51.6%

6. **Experiments rather than computational work were needed to prove that the additional 19 proteins harbor a PD fold if the authors decide to use this dataset for the gold standard.**

We manually looked at 123 false positive predictions (according to the labels of the Pires dataset) and found 78 that harbor a polysaccharide-degrading fold. We then go on to hypothesize that Pires *et al.* might have missed some depolymerases in their 2016 analysis. We have adapted our manuscript in the text and by providing the predicted protein structures of the 45 remaining false positive predictions in the Supplementary Data 1.

Lines 339-341:
"Manual investigations of the 45 remaining FPs revealed 32 proteins harboring a PD fold, suggesting that Pires et al. have missed some depolymerases in the phage genomes (Supplementary Table 8, Supplementary Data 1)."

7. **I have reservations about the practicality of predicting depolymerase activity at the amino acid level. Could the authors elaborate on how identifying domains contributes to predicting**

depolymerase activity at the protein level? Additionally, I am interested in how this approach might reveal new biological insights or enhance our understanding of depolymerase functions.

We agree with the reviewer that it is not exactly clear how identifying active domains contributes to accurately predicting depolymerase activity. The reason we constructed our model architecture in this way was not with the goal of improving the predictions at the protein level but rather because we wanted to go beyond what current tools can do and provide one single tool that could both annotate the protein as a whole and identify its enzymatic domain of interest. From our experience and through interactions with the research community, having both these functionalities in a single tool is highly valuable, for example, regarding depolymerase engineering purposes (Latka et al. 2021). Previous workflows would necessitate researchers to either manually look at predicted structures of the whole proteins or compare profile HMMs as a separate step. We have elaborated our discussion as follows:

Lines 368-372:
"We believe that predictions at this level can give a refined look at what exactly is being predicted by the model, especially when predictions are further investigated using 3D structure predictions. Consequently, such detailed predictions can improve and simplify depolymerase engineering workflows, which typically require multiple manual analysis steps (Latka et al. 2021)."

Minor comments

1. **Line 80: Please provide the full name of ESM-2.**

We have provided the full name of ESM-2 (*Line 83*).

2. **Line 93: Please provide the full name of CAZy.**

We have provided the full name of CAZy (*Line 97*).

3. **Line 176: Could you elaborate on the rationale for two convolutional layers followed by two full layers?**

The convolutional layers and the two forward layers were designed for the binary classification task. The pre-trained model outputs a list of labels assigned to each amino acid, which is then scrutinized for relevant patterns. The two forward layers process these distinct patterns, which were inferred during the training phase, to achieve the final classification.

Lines 237-240:
"Two convolutional layers identify relevant patterns in the list of labels assigned to each amino acid. These patterns are then processed by two forward neural network layers that output a prediction score reflecting how likely the protein sequence is a depolymerase."

4. **Line 179: What is the threshold of the prediction score? Is it 0.5?**

We have added the prediction score threshold (*Line 237*).

5. **Line 206: Please provide the full name of MCC.**

We have provided the full name of the MCC metric (*Lines 259-260*).

6. **Line 284: Could you explain why the ranking approach is not appropriate? And what do you mean by functionally annotating proteins? Is this the same thing as the binary classification?**

We have expanded our arguments on the ranking approach in the text:

Lines 397-400:
"We argue that this is not a particularly useful output format because it prevents making a single annotation prediction for each protein separately and hence necessitates comparing prediction scores across multiple proteins."

7. **Line 307: Why did you insist on manually adding the triple helix domain?**

Throughout our study, we used the prevalence of each fold as an indicator of its relevance to detecting depolymerases. However, the prevalence of the triple helix domain is not completely clear to us because we cannot easily detect its presence using structural analysis. This was in contrast with the low prevalence of the TIM β/α-barrel and α/α toroid domains, of which their removal would not significantly affect model performance. For this reason, we manually added sequences with a triple helix domain to ensure we did not overlook any important folds. We have clarified this in our manuscript text:

Lines 276-277:
"We annotated 146 triple helix domains manually and added these to the training set (Supplementary Table 6)."

8. **Line 329: What does the protein size mean? Why do you remove proteins under 200 amino acids in length?**

With protein size, we mean protein sequence length in terms of number of amino acids. We remove sequences smaller than 200 AAs because that is the minimum length above which we expect to find functional and complete depolymerase domains (as elaborated in *Latka et al. 2019*) . We have clarified these points in our text:

Lines 114-117:
"Protein sequences were then filtered according to their length, any sequence under 200 amino acids in length was discarded from the dataset, which is the minimum length above which we expect to find depolymerase domains (Pires et al. 2016; Latka et al. 2019)."

9. **Line 411: For training token classification and binary classification, why don't you use the same dataset?**

We decided not to use the same dataset for training token classification and binary classification modules based on (i) the fact that the modules were trained sequentially and (ii) the need to prevent the binary classifier from being exposed to sequences that the token classifier has already seen during its training. Using the same dataset for both modules could lead to biased outputs from the token classifier, as it

would produce outputs based on sequences that it has already seen, affecting the learning process of the binary classifier. Exposing the binary classifier to realistic outputs from the token classifier, allows for better generalization and more realistic learning outcomes.

10. **Line 433: Are the protein sequences predicted from phage genomes accurate enough? During the training process, you are using CDS.**

In both the training procedure and in the benchmark, we are using phage genomes collected from NCBI as raw data to start from. Our analyses are always done at the protein level, not at the DNA level, which might be confusing given our use of the term 'coding DNA sequence (CDS)'. We have adapted our phrasing in the text:

Lines 112-114:
"This collection comprised 24,289 entries of complete phage genome sequences, of which each protein sequence was pre-annotated via the PHROGS database, a protein resource specifically tailored for phage studies (Terzian et al. 2021)."

In 2016, Pires and colleagues focused on collecting every completely sequenced dsDNA phage. The extensive manual analysis that the authors performed on the genomes and their results give us great confidence about the general quality of the phage genomes and their associated proteins. In addition, we computed the number of called proteins per genome and their respective protein sequence lengths, which all fall within the range of what we typically expect for phages. Furthermore, if the quality of the protein data were unsatisfactory, we would expect both substantial false positives and false negatives across the different tools. At the same time, in the benchmark, we predominantly notice false positives (resulting in low precision).

11. **Line 435: Please provide formulas for computing recall, precision, F1, MCC, etc.**

We have provided the formulas for the computed performance metrics (*Lines 257-261*).

12. **Figure 2: Could you show the manually annotated 182 triple helix domains?**

We added Supplementary Table 6 with the data about the 146 annotated triple helix domains *(Lines 276-277)*.

13. **Figure 4: High resolution needed for its readability.**

We further improved the quality of Figure 4 and can upload the high-quality figure files separately at the end of the review process (for publishing).

14. **Table 1: Keep three or four significant figures for numerical values.**

We adjusted the numerical result values in Table 1 and Table 2.

Reviewer 2

Concha-Eloko et al. developed a language model-based ML method to accurately annotate phage depolymerases and their associated enzymatic domains. To be specific, the authors fine-tuned the language model ESM2 and trained two separate models tasked to predict token classification and binary classification, respectively. The manuscript is well-written overall, but the benchmark experiments have some design flaws. Although, the authors presented some results showing superior performance on binary classification task compared to DePP and PhageDPO on one dataset, the majority of the benchmark analysis is only self-benchmark and lacks baseline and other methods to compare with.

Major comments

The authors mentioned that phage depolymerases have high sequence diversity, but such features were not reflected by DepoScope. For both token and binary experiments shown by table 1, data was split randomly. The good performance using random split could be attributed to the similarity between training and testing set. To rule out similarity element and support the performance of DepoScope, the authors should consider split training and testing using clustering, so that sequences in training and those in test have low sequence similarity. The authors should also consider using cross validation to reduce randomness.

We appreciate the reviewer's fair concern. To avoid training and testing on identical and very similar sequences, we have clustered our collected protein sequences with CD-HIT at an identity threshold of 95% as part of our data processing and to reduce redundancy (*Lines 160-162*). To further accommodate the reviewer's feedback, we repeated model training and testing using protein sequence clustering at various identity threshold values (0.65, 0.70, 0.75, 0.80 and 0.85) to exclude similar sequences from the same (training or testing) group. From these additional experiments, we observe that the performance of these additional models does not vary significantly across the clustering thresholds, indicating that our model approach can generalize (Supplementary Table 8). We have extended our text in the Methods section:

Lines 242-246:
"In order to validate the ability of the model to generalize, the Group Shuffle Split method was used to split the data. Specifically, the sequences were clustered with CD-HIT before training using a range of clustering values (0.65, 0.70, 0.75, 0.80, 0.85). No significant impact on the performances was observed, indicating an ability to generalize on unseen data (Supplementary Table 5)."

With regard to the importance of cross-validation, we have a different view. Cross-validation is useful to extensively test models when only limited data are available and one would worry that a single train/test split would not be representative of the entire dataset. In that case, cross-validation is very useful to train and test across several splits and average those performances. However, we have collected 984 positive instances and 1,409 negative instances. Such numbers do not prevent a single test split from being representative as long as the sampling is random (the data is shuffled in advance).

For results in table 1, only self-benchmark is shown. The authors should consider including more baselines such as comparing with the traditional bioinformatics methods like BLASTp or using onehot encoding as a baseline to compare with LM embedding.

Although we understand where the reviewer is coming from with this remark, our view on such benchmarks differs from the reviewer's. First, several studies have already underscored the usefulness and superiority of protein language models for annotating proteins compared to BLASTp, including for viral proteins (Cantu et al., 2020, *PLOS Computational Biology*; Bileschi et al., 2022, *Nature Biotechnology*; Flamholz et al., 2023, *Nature Microbiology*). In particular, because viral proteins tend to have high genetic plasticity and evolve quickly, they are challenging to annotate with traditional alignment-based tools. For this reason, we do not consider BLASTp to be a relevant tool to benchmark our method against. We have clarified this in the text:

Lines 56-57:
"For this reason, depolymerases are typically difficult to identify using alignment-based methods (Cantu et al. 2020; Flamholz et al. 2023)."

Here, we wanted to innovate beyond the state-of-the-art in two ways: (1) use a protein language model to specifically annotate phage depolymerases and (2) provide annotations at the level of individual amino acids (the token-level classification). In particular, we decided to build on top of the ESM-2 models because of their public availability and ease of use. We do not claim that this approach is the absolute best approach to predict such annotations, rather we demonstrate its relative superiority to other tools specifically designed for depolymerase annotation. This, in our view, is the most relevant comparison. We could have compared our method (building on top of ESM-2) with many other methods (including one-hot encodings but also more traditional protein descriptors such as physicochemical parameters, *k*-mer patterns and more). We believe that exploring such other methods would be relevant only when our chosen approach to leverage ESM-2 would bring about unsatisfactory results. Because this is not the case, we have not considered training and comparing against other methods.

What does "DepoScope correct" mean in table 2?

With "DepoScope corrected", we were referring to the results of DepoScope when it was corrected for the false positive predictions in the Pires dataset that nevertheless presented a PD domain after manual inspection. We have adjusted the expression in our manuscript text for better clarity:

Lines 344-345:
"When only considering the remaining 13 proteins as FPs, the MCC of DepoScope increases to 74.4% (referred to as Deposcope FP adjusted)."

Minor comments:
2 convolutions followed by two dense layer is rarely called a "decoder" structure, as "decoder" usually implies the upsampling blocks that reconstruct a bottleneck layer.
We thank the reviewer for this remark. Indeed, the terms "encoder" and "decoder" could be misleading. We referred to the fine-tuned ESM-2 model for token classification as the encoder part, while the two convolution layers and dense layers were referred to as the decoder. To enhance clarity, we have adjusted these terms throughout the manuscript text.

Lines 87-90:
"We developed a new machine learning tool called DepoScope consisting of a fine-tuned ESM-2 model, followed by a set of convolutional and dense neural network layers to both detect depolymerase protein sequences and precisely identify the location of their enzymatic domains."

Lines 208-210:
"DepoScope is built as a stacked model architecture with two parts (Figure 3). The first part is a fine-tuned ESM-2 model for a token classification and the second part is a pair of two convolutional layers and two forward neural layers for a binary classification task."

Please double check the citations, for example, Page 19 line 547, the DOI for DePP is wrong.
We have manually double-checked and corrected our References. We had no issues opening the DOI link for the DePP paper, although some DOI links could not be opened using all browsers.

How are prediction cutoff/threshold determined by DePP and PhageDPO on Pires dataset? The precision of both models is awfully low, but recalls are really high, which seems like the cutoff/threshold is too low. Area under precision-recall curve might serve as a better metrics.
We thank the reviewer for this insightful suggestion. In fact, none of the DePP or PhageDPO studies cover details of an appropriate cutoff on the prediction score for their tools. For this reason, we worked with a standard cutoff of 0.5 for all of the tools. As the reviewer suggests, the PR AUC metric indeed seems appropriate as a cutoff-independent metric to assess performance. Therefore, we have computed the PR AUC for each benchmarked tool and added the results to Table 2. For completeness, below we have also appended the PR curves in a figure. We have accordingly extended our results in the text:

Lines 304-306:
"We computed six performance metrics (at a prediction threshold of 0.5 when relevant): precision (P), recall (R), accuracy, F1 score, MCC and the PR area-under-the-curve (Table 2)."

Lines 331-333:
"Overall, DepoScope achieves a PR AUC of 42.3%, compared to 10.2% and 27.8% for DePP and PhageDPO, respectively."

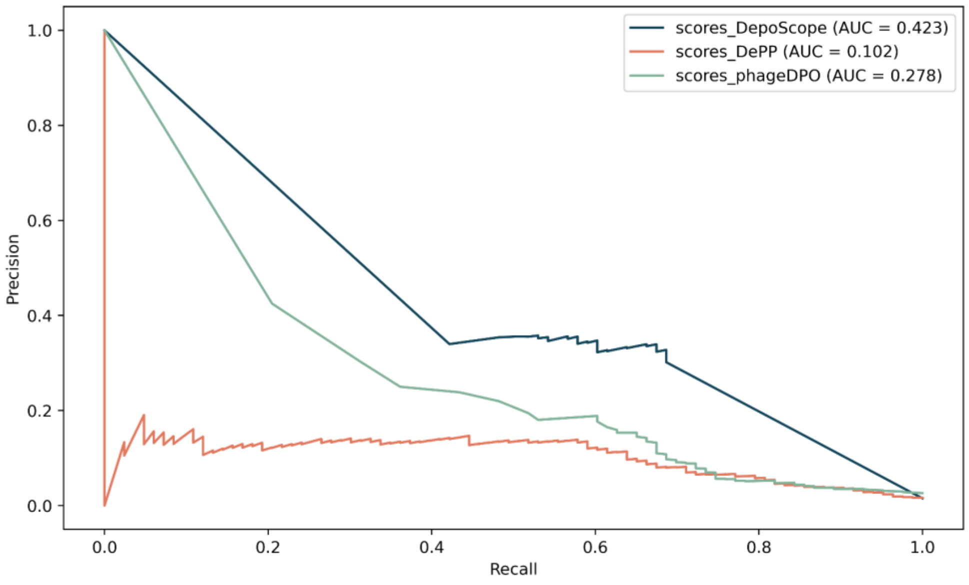

Figure: Precision-Recall curves for the benchmarked tools, together with their area-under-the-curve.

Reviewer 3

This paper presents an approach to annotating phage depolymerases by leveraging a fine-tuned ESMFold model, supplemented with two 1D convolutional layers and a dense layer. The writing is well-crafted, and the code is user-friendly.

We thank the reviewer for the positive words about our work.

However, it's important to note the presence of established tools for annotating the Enzyme family utilizing deep learning techniques: https://www.ncbi.nlm.nih.gov/pmc/articles/PMC10634757/. The task of annotating phage depolymerases, as described, appears to be more straightforward than the comprehensive annotation required for the Enzyme family, primarily involving binary classification. This simplification could potentially limit the scope of the tool's applicability and innovation.

It is great for the reviewer to suggest a related (preprint) study that is relevant for us to include in our manuscript. The method developed in the study, however, is different in its focus on binary and multiclass classification, while our method first does a token classification, followed by a binary classification. We have adapted our text accordingly:

Lines 97-99:
"A recently developed tool can classify CAZymes broadly but is not specifically tailored towards detecting phage depolymerases (Thurimella et al., 2023)."

In addition, this paper is lack of downstream analysis, like substrate prediction.

To our current knowledge, substrate prediction at the level of individual depolymerases is beyond the scope of what is currently possible today due to the lack of sufficient experimental data at that detailed level in combination with the tremendous substrate diversity (driven by the intense phage-host co-evolution process). While there are smaller, high-quality experimental datasets available (e.g. https://pubmed.ncbi.nlm.nih.gov/34527194/), these datasets are too small to develop comprehensive machine learning tools off of. The current state-of-the-art tools are (only) able to predict interactions between a collection of receptor-binding proteins and corresponding receptors (https://www.researchsquare.com/article/rs-3101607/v1).
