# Supplementary material for: DepoScope: Accurate phage depolymerase annotation and domain delineation using large language models
Source: PLoS Comput Biol. 2024 Aug 5;20(8):e1011831. doi: 10.1371/journal.pcbi.1011831 (PMC11326577; doi:10.1371/journal.pcbi.1011831)
Supplement: Attachment — Submitted filename: RCE_Deposcope_response2.pdf [file pcbi.1011831.s006.pdf]

Response to reviewers

DepoScope: accurate phage depolymerase annotation and domain delineation using large language models

We sincerely thank the reviewers for providing us with additional feedback to further improve our work. Their comments and our point-by-point responses to address their comments are elaborated below.

Reviewer #1

The authors have effectively addressed the comments. The article can now be considered for acceptance.

We thank the reviewer for this positive assessment of our submitted revision.

Reviewer #2

Overall, the authors have addressed most of my comments. I appreciate the authors' efforts.

We thank the reviewer for these kind words on our work.

For my first major comment, 0.65 sequence identity is still relatively high. Can the authors show the result under 0.3 clustering? Since above 0.3, the sequences can still be considered as homologous.

We agree with the reviewer that a sequence threshold of 0.65 is still relatively high when it comes to protein function annotation. The threshold of 30% is indeed a typical limit for (sequence) homology, as described by Rost, 1999 (https://pubmed.ncbi.nlm.nih.gov/10195279/). However, Rost then also described that at lower thresholds for sequence identity (<30-35%), structural similarities also become blurred. Given our focus on conserved structural features in phage depolymerases, we have therefore evaluated the model predictions in a grouped setting across lower thresholds until 30% sequence identity. We observe that the models maintain high performances for the metrics used across the tested sequence identity thresholds (Table below).

Threshold value	precision	recall	specificity	accuracy	f1	mcc
0.25	0.9889	0.9889	0.9929	0.9913	0.9889	0.9817
0.3	0.9886	0.9667	0.9929	0.9826	0.9775	0.9635
0.35	0.9888	0.9778	0.9929	0.987	0.9832	0.9726
0.4	0.9888	0.9778	0.9929	0.987	0.9832	0.9726
0.45	0.9888	0.9778	0.9929	0.987	0.9832	0.9726

We adapted the corresponding sentences in our manuscript text:

Lines 245 – 248:
"*Specifically, the sequences were clustered with CD-HIT before training using a range of clustering values (0.25, 0.30, 0.35, 0.40, 0.45, 0.65, 0.70, 0.75, 0.80, 0.85). No significant impact on the performances was observed, indicating an ability to generalize on unseen data (Supplementary Table 5).*"

For my second major comment, I understand that BLASTp may not be the best benchmark to use. However, in authors' response I did not see a strong reason for not using one-hot encoding as a benchmark. Without benchmarking with comparing to one-hot encoding it is hard to claim the superiority of your model is due to ESM2 embedding.

We understand that the reviewer is revisiting this issue, but we respectfully disagree. In our work, we do not claim that our results are superior specifically because we use ESM-2 embeddings. Instead, we have developed a novel, ESM-2 based method that we claim (and subsequently show) is better than other available tools for depolymerase detection and can, in addition, make predictions at the level of individual amino acids. We have chosen to build on top of the ESM-2 models because of their open-source availability, ease-of-use and potential further improvements in the future, which are reasons we clearly mention in the manuscript (*Lines 362–367*). More importantly, our approach reaches satisfactory performance. Given this satisfactory result, and because we do not claim that our approach is better specifically because of the ESM-2 embeddings, we cannot see any reason why our choice to not compare our approach against one-hot embeddings would mean that our work is lacking in quality. We hope the reviewer understands our point of view and we invite any further feedback on this.

We have rephrased the following sentence in the Availability and Future Directions section that we think might cause confusion (specifically, removing 'best-in-class' from the sentence).

Lines 354 – 356:
"Our work goes beyond the current state-of-the-art in depolymerase annotation by approaching data collection and processing in a novel and comprehensive way, followed by leveraging the ~~best-in-class~~ protein language model ESM-2 (Lin et al. 2023)."
