# Supplementary material for: DepoScope: Accurate phage depolymerase annotation and domain delineation using large language models
Source: PLoS Comput Biol. 2024 Aug 5;20(8):e1011831. doi: 10.1371/journal.pcbi.1011831 (PMC11326577; doi:10.1371/journal.pcbi.1011831)
Supplement: Attachment — Submitted filename: Review_DepoScope.docx [file pcbi.1011831.s004.docx]

In this study, the authors introduce DepoScope, an innovative machine-learning tool to provide an accurate annotation and domain identification of phage depolymerases. They developed DepoScope using the cutting-edge, pre-trained ESM2 protein language model and a two-layer convolutional neural network. To better train the model, the authors collected and processed a comprehensive set of protein sequences. The findings enhance our understanding of phage depolymerases, especially at the amino acid level. However, some major and minor issues still need to be addressed.

**Major comments**

1. The authors have claimed that their model surpasses two other recently developed depolymerase detection tools, DePP and PhageDPO, in performance as evidenced by the benchmark using the dataset from Pires *et al.* My primary concern centers on whether this superior performance of DepoScope is attributed to the curated dataset used for its training or to the inherent strengths of its model architecture. This leads to a crucial question: if DePP and PhageDPO were trained using the same dataset as DepoScope, would their performance metrics, specifically in terms of F1 score and MCC, align closely with those of DepoScope?
2. The authors have noted that previous studies may have been constrained by “small and undiversified” datasets. However, upon examining the PhageDPO more closely, its training data comprises 1,437 positive sequences from NCBI, encompassing six depolymerase-associated domains. Could the authors kindly provide a comparison between the dataset used in their study (1,926 positive sequences) and that used by PhageDPO?
3. Data imbalance presents a significant challenge in classification tasks. Could the authors consider experimenting with varying numbers of negative controls in their training dataset, similar to the approach taken by PhageDPO, to see how these alterations impact the overall model performance?
4. The authors’ decision to exclude certain fold types due to their limited occurrence is understandable. However, in the interest of enhancing the model’s generalizability, would it be feasible for the authors to classify folds like the TIM β/α-barrel, and α/α toroid folds under an “others” category?
5. The substantial difference in MCC between DepoScope and the other two models was quite striking. This leads me to question whether the training data for DepoScope included the dataset from Pires et al., especially considering that INPHARED downloads and filters phage genomes from GenBank. For clarity and thorough understanding, it would be beneficial if the authors could explicitly state whether the Pires et al. dataset was incorporated into the training data.
6. Experiments rather than computational work were needed to prove that the additional 19 proteins harbor a PD fold if the authors decide to use this dataset for the gold standard.
7. I have reservations about the practicality of predicting depolymerase activity at the amino acid level. Could the authors elaborate on how identifying domains contributes to predicting depolymerase activity at the protein level? Additionally, I am interested in how this approach might reveal new biological insights or enhance our understanding of depolymerase functions.

**Minor comments**

1. Line 80: Please provide the full name of ESM-2.
2. Line 93: Please provide the full name of CAZy.
3. Line 176: Could you elaborate on the rationale for two convolutional layers followed by two full layers?
4. Line 179: What is the threshold of the prediction score? Is it 0.5?
5. Line 206: Please provide the full name of MCC.
6. Line 284: Could you explain why the ranking approach is not appropriate? And what do you mean by functionally annotating proteins? Is this the same thing as the binary classification?
7. Line 307: Why did you insist on manually adding the triple helix domain?
8. Line 329: What does the protein size mean? Why do you remove proteins under 200 amino acids in length?
9. Line 411: For training token classification and binary classification, why don’t you use the same dataset?
10. Line 433: Are the protein sequences predicted from phage genomes accurate enough? During the training process, you are using CDS.
11. Line 435: Please provide formulas for computing recall, precision, F1, MCC, etc.
12. Figure 2: Could you show the manually annotated 182 triple helix domains?
13. Figure 4: High resolution needed for its readability.
14. Table 1: Keep three or four significant figures for numerical values.
